# Low-Dose Elemental Mapping of Light Atoms in Liquid-phase Materials Using Cryo-EELS

**DOI:** 10.1021/acs.analchem.5c02121

**Published:** 2025-07-31

**Authors:** Daisuke Unabara, Yohei K. Sato, Tasuku Hamaguchi, Koji Yonekura

**Affiliations:** † Institute of Multidisciplinary Research for Advanced Materials (IMRAM), 13101 Tohoku University, 2-1-1 Katahira, Aoba-ku, Sendai, Miyagi 980-8577, Japan; ‡ Biostructural Mechanism Laboratory, RIKEN SPring-8 Center, 1-1-1 Kouto, Sayo, Hyogo 679-5148, Japan

## Abstract

Cryogenic transmission electron microscopy (cryo-TEM) enables the visualization of liquid-phase materials, including nanoparticles and soft/biomaterials, under cryogenic conditions while minimizing radiation damage. Cryo-TEM imaging provides insights into particle size, shape, and dispersion. Beyond such conventional structural information, acquiring elemental composition data allows for a more detailed analysis and evaluation. In this study, we developed a method for elemental mapping of nanoparticles and soft/biomaterials in frozen solvent by integrating electron energy loss spectroscopy (EELS) with energy-filtered (EF) cryo-EM. Cryo-EELS and analysis were performed using the three-window method, following the alignment and correction of stage drift during image acquisition and interexposure drift between different energy windows. This approach, by balancing accurate and reliable signal extraction with electron dose, enabled the generation of elemental maps for nanoparticles as small as 10 nm in frozen solvent. Furthermore, we extended the technique to protein-coated silica nanoparticles and hydroxyapatite (HAp) nanoparticles in vitrified solvent, selecting specific energy loss values to identify multiple constituent elements. As a result, we successfully mapped silica from the nanoparticle cores, carbon from the protein shell of the nanoparticles, and phosphorus and calcium—key light elements in biological systems—within the same imaging area of the HAp particles.

## Graphical abstract

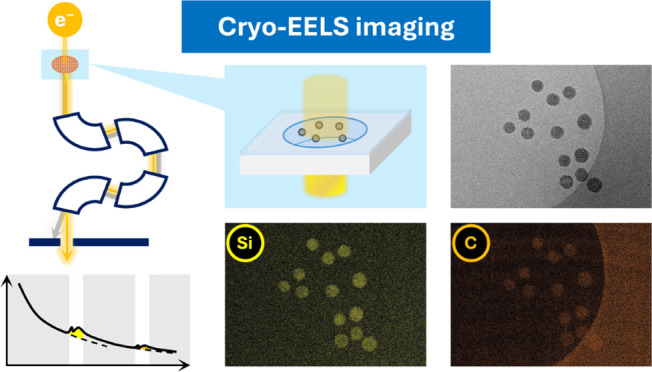

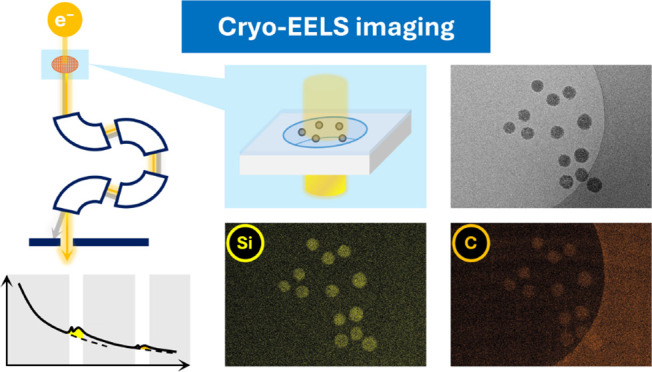

## Introduction

Understanding the structural and chemical properties of liquid-phase materials is crucial for advancing research in fields such as nanotechnology, biomaterials, and soft matter science. Cryogenic transmission electron microscopy (cryo-TEM) has become a powerful tool for the determination of high-resolution three-dimensional structures of proteins and protein complexes frozen in vitreous ice,^1,2^ via single-particle analysis.^3–8^ Cryo-TEM minimizes electron beam damage while preserving biological specimens in a near-native state.^9^ Recently, cryo-TEM has been applied to nonbiological materials, such as nanoparticles, micelles, vesicles, nanotubes, and supramolecular polymers, offering insights into their size, morphology, and dispersion state.^10–16^ However, determining the elemental composition of these materials directly from cryo-TEM images remains challenging.

Elemental mapping techniques like energy-dispersive X-ray spectroscopy (EDS) and electron energy loss spectroscopy (EELS) are used in electron microscopy. EELS, in particular, provides high sensitivity to light elements and enables the analysis of chemical bonding states, making it an excellent tool for nanoscale elemental analysis. When combined with energy-filtered transmission electron microscopy (EF-TEM), EELS imaging allows for the mapping of elemental distributions within a sample.^17–20^ However, conventional EELS imaging requires a high electron dose, which causes significant damage, making it less suitable for radiation-sensitive samples.^21–25^ Typical cryo-EM imaging utilizes elastically scattered electrons, while interactions between incident electrons and the sample also generate inelastically scattered electrons with a broad range of energy losses. These energy-loss electrons contain weak signals from inner-shell electron excitations of specific elements and chemical bonds, but the signals are not effectively utilized. In fact, the application of elemental mapping to frozen-hydrated samples has so far been limited to heavy metal particles approximately 20 nm in size,^26^ and to intracellular regions ranging from several hundreds of nanometers to 1 μm^27^

To address this limitation, we developed a method that applies parallel illumination of a coherent beam at low dose rates for cryo-EELS imaging, combined with the three-window method^28^ for image analysis incorporating image drift corrections.^29–31^ We demonstrated that this approach enables accurate and reliable mapping of multiple elements in nanoparticles and biomaterials dispersed in frozen solvent while balancing with electron dose, significantly enhancing the potential of cryo-TEM for materials science research. It will offer a more comprehensive characterization of complex liquid-phase materials, such as hybrid materials and nanomaterials with organic modifications dispersed in solvent.

## Experimental Section

### Materials

Unmodified silica nanoparticles of 10, 30, and 50 and 100 nm streptavidin-coated silica particles with a biotin-binding capacity of >5 pmol/mg were purchased from Micromod. Stock solutions were prepared at a concentration of 25 mg/mL in water for the 10, 30, and 50 nm particles and PBS containing 0.02% sodium azide for the streptavidin-coated particles, then diluted with water to a final dispersion of 2.5 mg/mL for EELS imaging. For EELS spectral measurements, the stock solution of the streptavidin-coated particles was used without further dilution. Hydroxyapatite (HAp) was purchased from Merck and dispersed in water from its paste form to prepare a 2.5 mg/mL suspension. The solution of the streptavidin-coated particles was kept on ice, while all the other samples were stored at 20 °C until use.

### Cryo-TEM Sample Preparation

Copper grids with 200 mesh, covered with holey carbon film (Quantifoil R1.2/1.3), were hydrophilized in advance using a glow discharge device (JEC-3000FC, JEOL). A 3 μL aliquot of the nanoparticle-containing sample solutions was applied to each grid. The grids were then blotted with filter paper under 95% humidity and at 4 °C and rapidly vitrified by plunging into liquid ethane using a semiautomatic EM GP2 plunger (Leica Microsystems). The prepared grids were stored in liquid nitrogen until cryo-TEM analysis.

### EELS Spectral Measurement

The vitrified grids were transferred to a CRYO ARM 300 II electron microscope (JEOL), equipped with a cold field-emission gun (CFEG) and an in-column omega filter, and examined at an accelerating voltage of 300 kV. The CRYO ARM microscope employs a dedicated specimen cartridge instead of standard cryo holders. The specimen stage was cooled to 89 ± 1 K via thermal conduction from a liquid nitrogen tank, and temperature fluctuations were negligible. Consequently, stage drift due to temperature variation was minimal (drift rate <2 nm/min). The observed specimen drift was attributed primarily to beam-induced motion, in addition to minor stage drift.

EELS spectra were acquired in spectrum mode using an XF416ES detector (TVIPS), which offers high tolerance to electron beam exposure and a wide dynamic range, after positioning the region of interest (ROI) in Mag mode at 60k magnification with a 20 μm objective aperture. For nanoparticle measurements, the ROI was adjusted within amorphous ice in a hole, excluding the carbon support film using a 20 μm selected-area (SA) aperture. The XF416ES detector was set to high-sensitivity mode, and each spectrum was acquired using the EM-Menu software. First, the zero-loss spectrum was recorded with an exposure time of 63 ms, corresponding to a dose of 1.2 e^–^/Å^2^. Subsequently, an energy shift (ES) to the absorption edge of each element was introduced by applying an additional voltage to the electron gun using a newly developed accessory program, EnergyShift (Figure S1), included in the ParallEM package.^32–34^ Core-loss spectra were then acquired with exposure times of 10 s for carbon from supporting film, 40 s for carbon from the protein coating on silica nanoparticles, 20 s for oxygen from solvent ice, 1–2 s for silicon from nanoparticles, 1 s for phosphorus from HAp, and 3 s for calcium from HAp. Spectral intensity was summed over a width 101 pixels across the energy loss spectrum using EM-Menu (Figure S2) to obtain all spectral graphs shown in the figures. The measurement conditions are summarized in Table 1.

**Table 1:** Experimental Parameters for Measuring Cryo-EELS Spectra

	concentration (mg/mL)	magnification	exposure (s)		total exposure (s)	dose rate (e^–^/Å^2^/s)	total dose (e^–^/Å^2^)
			zero-loss	core-loss edge			
carbon from carbon film		60k	0.063	10	30	19.3	579
oxygen from vitrified ice		60k		20			
silicon from 50 nm nanoparticles	2.5	60k	0.063	2	2	20.3	40.6
silicon from 100 nm nanoparticles	25	60k	0.063	1	41	21.8	894
carbon from streptavidin		60k		40			
phosphorus from HAp	2.5	60k	0.063	1	4	20.3	81.2
calcium from HAp		60k		3			

### Cryo-EELS Imaging

For element mapping, the three-window method^28^ was used, capturing three images of a single ROI at three energy windows (prepre edge, pre edge and core loss; Figures 1 and S3; see below). EELS and zero-loss images were acquired using a direct electron detection camera,^35,36^ K3 (AMETEK Gatan), which features high sensitivity and a high frame rate, with an energy slit width of 15, 20, or 40 eV. The magnification was set to 4k with a dose rate of 0.8 e^–^/Å^2^/s for detecting the carbon and oxygen signals from the carbon film and vitrified water, and to 25k with a dose rate of 8.0–8.3 e^–^/Å^2^/s for elemental mapping of silica and HAp nanoparticles. SerialEM^37^ was used for imaging area search, focus adjustment, and ROI alignment for each energy window, while DigitalMicrograph was used for final data acquisition with drift corrections during exposure.

**Figure 1:**
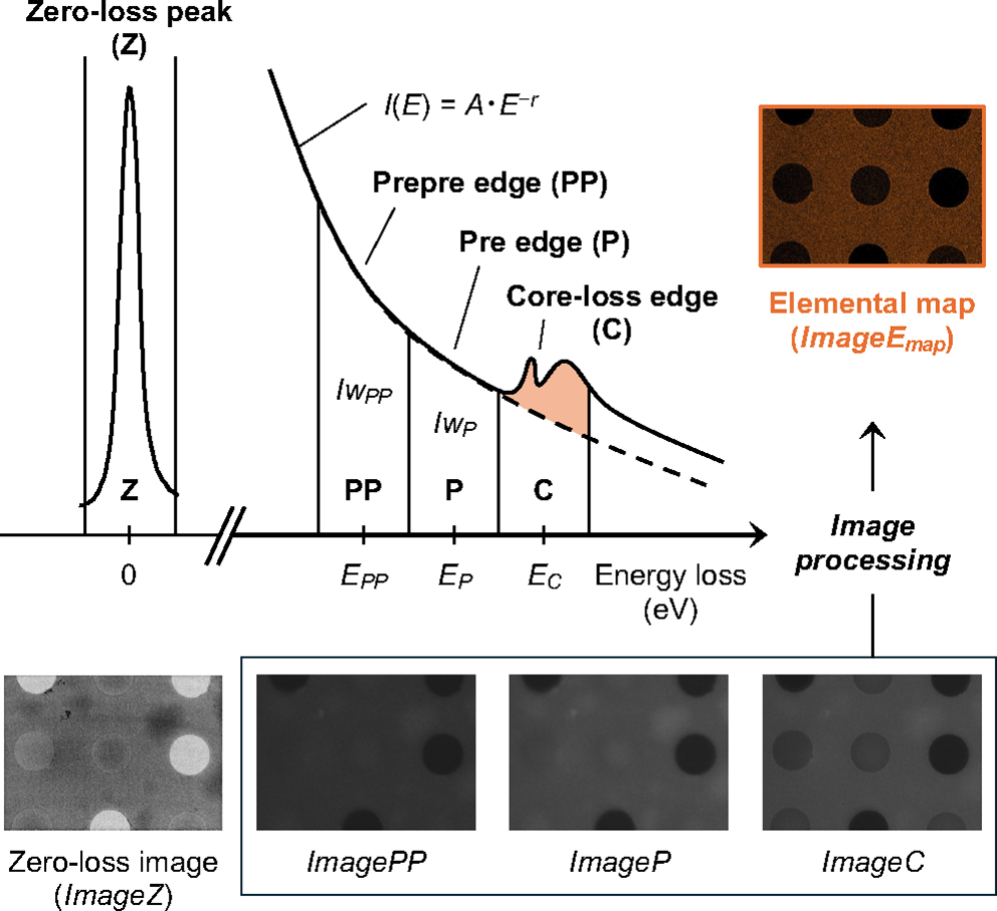
Schematic depiction of the elemental mapping using the three-window method. *Iw*_*PP*_ and *Iw*_*P*_ represent the summations of intensity within the prepre-edge window and pre-edge window, respectively, and *E*_*PP*_, *E*_*P*_ and *E*_*C*_ correspond to the central energy levels for the associated windows. The background intensity in *ImageC* was estimated from the attenuation curves of *ImagePP* and *ImageP*, resulting in the elemental map, *ImageE*_*map*_, as shown in eqs 1–4.

The imaging procedure was as follows:

1. The stage was moved to the target area in View mode in SerialEM at ×8k magnification.
2. In Focus mode, the defocus was set to approximately −4 μm to acquire the first zero-loss images of carbon film and vitrified water and −1 μm to acquire those of the silica and HAp nanoparticles.
3. The imaging condition was set to Recode mode in SerialEM. In this mode, the shutter remained closed to prevent unnecessary damage to the samples, except for the specified exposures specified below.
4. A zero-loss image was acquired using DigitalMicrograph with an exposure time of 0.5 or 1.0 s. An additional short exposure was also applied in SerialEM to acquire a zero-loss image, which served as a template for image alignment across different energy windows.
5. Defocus was adjusted to 0 μm, and ES was adjusted to the prepre-edge energy loss value using EnergyShift. Images were acquired with exposure times of 1 and 4 s, corresponding to total doses of 0.8 and 3.2 e^–^/Å^2^, for carbon and oxygen from the vitrified holey carbon grid, respectively (Table 2). Dose-fractionated images were acquired with exposure times of 4–6 s, corresponding to total doses of 32–45.6 e^–^/Å^2^, for carbon, silicon, phosphorus, and calcium from the nanoparticles (Table 2). All frames were aligned to correct stage drift and summed to enhance signal intensity.
6. ES was reset to 0 eV, and a short-exposure zero-loss image was recorded using SerialEM at a defocus of 0 μm. The AlignTo command in SerialEM was used to align the ROI via image shift by correlating with the saved template image.
7. ES was set to the pre-edge energy loss value, and images were acquired at a defocus of 0 μm with exposure times of 5 and 20 s, corresponding to total doses of 4 and 16 e^–^/Å^2^, for carbon and oxygen from vitrified holey carbon grid, respectively and dose-fractionated images were acquired at a defocus of 0 μm with exposure times of 20–30 s, corresponding to total doses of 160–228 e^–^/Å^2^, for carbon, silicon, phosphorus, and calcium from the nanoparticles (Table 2).
8. After aligning the ROI to the zero-loss image, ES was set to the energy loss value of the core-loss spectrum, and dose-fractionated images were acquired with the same exposure times as in the pre-edge exposure.
9. Finally, after aligning the ROI, an additional zero-loss image was acquired to evaluate the sample condition before and after all steps.

For each acquired image, the background intensity of the core-loss image (*ImageC*) was estimated using the prepre-edge image (*ImagePP*) and pre-edge image (*ImageP*), and elemental maps (*ImageE_map_* s) were generated using DigitalMicrograph Version 3.61.

The spectral intensity, *I*, decays with energy loss (*E*) following a power law, as given by *I*(*E*) = *A*·*E* ^–*r*^,^28^ where *A* and *r* are fitting parameters. This curve can be used to estimate the background level of the core-loss signals, and *r* can be derived as follows

1 $$r=\frac{\log{Iw}_{P}-\log{Iw}_{PP}}{\log\left(E_{PP}/E_{P}\right)}$$

where *Iw*_*PP*_ and *Iw*_*P*_ represent the summations of intensity within the prepre-edge window and pre-edge window, respectively, and *E*_*PP*_ and *E*_*P*_ correspond to the central energy-loss values for the associated windows. The same window widths were used for both *Iw*_*PP*_ and *Iw*_*P*_, set to 15 for phosphorus and calcium, 20 for oxygen and silicon, and 40 eV for carbon. Here, the relationship between each data acquisition window is given by

2 $$A=\frac{{Iw}_{PP}}{E_{PP}^{-r}}=\frac{{Iw}_{P}}{E_{P}^{-r}}$$

The background constant in the core (*k*) is estimated from the prepre-edge and pre-edge windows as

3 $$k={\left(\frac{E_{C}}{E_{P}}\right)}^{-r}$$

Finally, the elemental map or the energy-loss (EL) image (*ImageE*_*map*_) is generated by subtracting the background from the core-loss image (*ImageC*)­

4 $$ImageE_{map}=ImageC-k\cdot ImageP$$

The calculations in eqs 1–4 can be performed using a self-made DigitalMicrograph script, EFImageGenerator. The imaging conditions are summarized in Table 2.

**Table 2:** Experimental Parameters for Acquiring Cryo-EL Images

	magnification	pixel size (nm)		exposure (s)		total exposure (s)	dose rate (e^–^/Å^2^/s)	total dose (e^–^/Å^2^)	slit width (eV)
			zero-loss	prepre edge	pre edge and core-loss edge				
carbon from carbon film	4k	1.29	0.5	1	5	11	0.8	8.8	40
oxygen from vitrified ice	4k	1.29	0.5	4	20	44	0.8	35.2	20
silicon from 10 nm nanoparticles	25k	0.21	1	6	30	66	7.6^a^	502^a^	20
silicon from 30 and 50 nm nanoparticles	25k	0.21	0.5	4	20	44	7.6^a^	334^a^	20
silicon from 100 nm nanoparticles	25k	0.21	0.5	4	20	44	8.0	352	20
carbon from streptavidin	25k	0.21	0.5	4	20	44	8.0	352	40
phosphorus from HAp	25k	0.21	1	5	25	55	8.3	457	15
calcium from HAp	25k	0.21	1	5	25	55	8.3	457	15

a Measured through the specimen, while the others were measured without the specimen.

### Estimation of Particle Size Distribution

The particle-size distribution of silica nanoparticles was estimated using Fiji^38^ as follows: Image contrast was enhanced by applying 4 × 4 binning, median filtering, and background subtraction, followed by 8 bit conversion and binarization. Some of the aggregated particles were segmented using the watershed algorithm. The processed images were then analyzed to obtain size information for the major and minor axes based on area and circularity.

### Image Processing for Images of 10 nm Silica Nanoparticles

Image processing for 10 nm silica nanoparticles was performed using Fiji as follows: image contrast was enhanced by applying 8 × 8 binning, Gaussian blurring with a sigma value of 1.0 radius for the zero-loss image and 2.0 radius for the EL image. The zero-loss image was then inverted, and both the inverted zero-loss image and the EL image were converted 8 bit and subsequently binarized. The binarized zero-loss image and EL image were superposed to evaluate the detection of silicon atoms.

The thickness (*t*) of the ice layer was estimated from the zero-loss image intensity (*I*_+ef_) and unfiltered intensity (*I*_–ef_) as

$$t=\Lambda \cdot \ln\left(I_{-ef}/I_{+ef}\right)$$ 5

where *Λ* is the mean free path of inelastically scattered electrons, assumed to be approximately 400 nm for 300 kV electrons.^39^

## Results and Discussion

### Spectral Analyses and Imaging of Carbon and Water in Vitrified Grid

Conventional cryo-TEM analysis relies solely on elastically scattered electrons and can gain image contrast by removing inelastically scattered electrons.^39^ In contrast, cryo-EELS utilizes inelastically scattered electrons that lose specific levels of energy due to interactions within the sample. The presence of elements can be analyzed in energy spectrum mode, and selecting specific spectral ranges enables elemental mapping in imaging mode of EF-TEM. This study applied a standard cryo-EM setup for high-resolution single-particle analysis of proteins and three-dimensional electron diffraction of small crystals,^32–34^ to conduct cryo-EELS measurements.

We first acquired EELS spectra from vitrified ice on a holey carbon grid at 60k magnification, where a thin layer of frozen water was distributed over the carbon supporting film and holes. A spectrum obtained at zero ES exhibited a zero-loss peak along with a low-energy loss region, including plasmon signals (Figure 2a). Subsequent spectral measurement at an energy loss of 295 eV corresponding to the carbon (C)–K edge revealed a C–K edge signal originating from the amorphous carbon in the support film (Figures 2b and S4). Further applying an ES of 535 eV, corresponding to the oxygen (O)–K edge, enabled the detection of the O–K edge signal from amorphous ice (Figures 2c and S4).^40^ Following spectrum acquisition, elemental mapping was performed using the three-window method, in which the background was estimated from the prepre-edge and pre-edge images and subtracted from the core-loss image as in eqs 1–4. In this process, image alignment between different energy ranges was omitted, as the image drift was not significant. In the zero-loss image acquired at 4k magnification, a thin ice film was clearly visible within the regularly arranged holes (Figure 2d), along with residual ice deposits on the carbon support film. Within the same ROI, elemental mapping based on the C–K edge and O–K edge signals revealed that the C–K edge signal was localized to the carbon support film (Figure 2e), whereas the O–K edge signal was detected across the support film and the ice-filled holes (Figure 2f). These images were also analyzed using the two-window method, where *ImageP* corresponding to the pre-edge window was simply subtracted from *ImageC* corresponding to the core-loss window to generate *ImageE*_*map*_. This produced similar images, but with slightly higher backgrounds particularly in the holes on the right side of the C–K core-loss image (Figure S5a,b).

**Figure 2:**
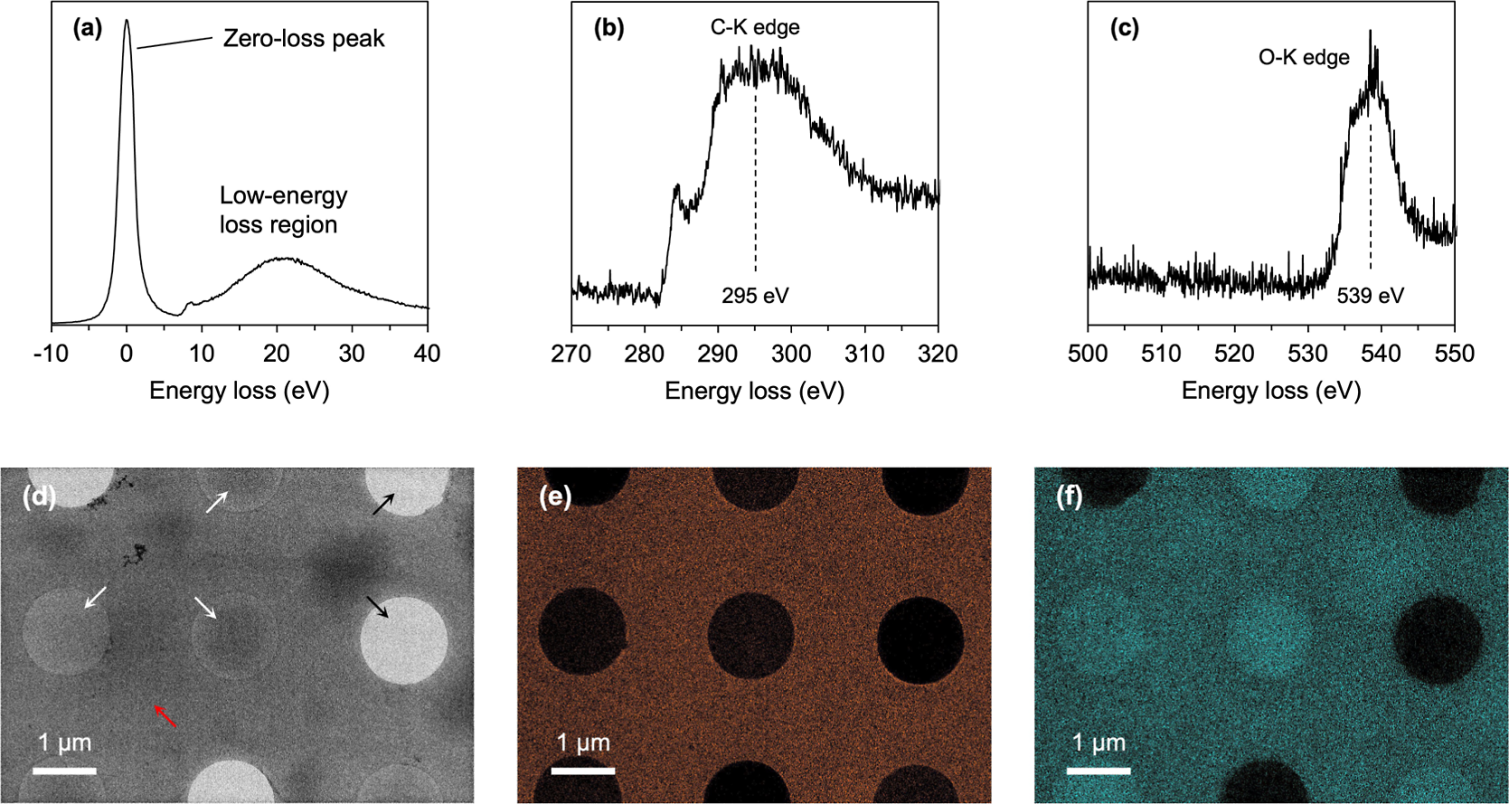
Cryo-EELS and elemental mapping of carbon and oxygen from vitrified holey carbon grid. (a) EELS spectrum (zero-loss peak and low-energy loss region). (b) EELS spectrum of the C–K edge. (c) EELS spectrum the O–K edge. (d) Zero-loss image. White and black arrows indicate holes with and without vitrified ice, respectively. Red arrow indicates a thin layer of vitrified ice on carbon support film. (e) Cryo-EL image of the C–K edge. The C–K core-loss signals are shown in orange. (f) Cryo-EL image of the O–K edge. The O–K core-loss signals are shown in cyan.

### Elemental Mapping of Silica Nanoparticles

To explore potential applications in materials science, we examined the detection of silicon-derived EELS spectra and the elemental mapping of silica nanoparticle dispersions. Silica dispersions were prepared in amorphous ice on a holey carbon grid (Figure 3a). In conventional EELS analysis, SiO_2_ particles typically exhibit Si^0^ and Si^4+^ signals within the 100–110 eV range, as previously reported.^41^ EELS spectra acquired for 50 nm silica nanoparticles frozen within the holes (Figure 3b,c) confirmed the detection of the Si-L_2,3_ core-loss signal at approximately 105 eV (Figure 3d), consistent with previous observations in dried samples. However, in cryo-EELS spectra acquired from frozen solvent samples, the dominant plasmon signal from water partially obscured the silicon signal, highlighting the challenges associated with elemental detection in hydrated environments.

**Figure 3:**
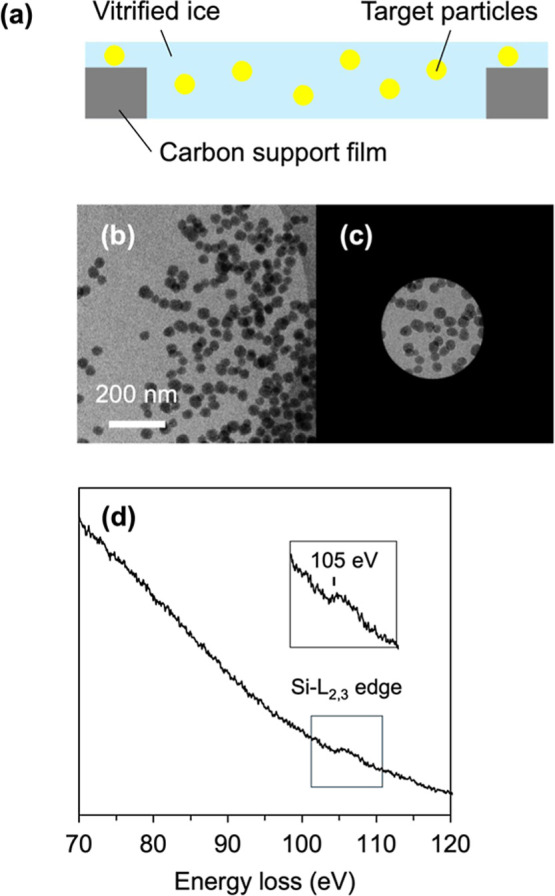
EELS spectrum from dispersed silica nanoparticles in vitrified ice. (a) Schematic depiction of the side view of nanoparticles within vitrified ice. (b) Zero-loss image of dispersed nanoparticles in amorphous ice. (c) Selected area for EELS spectrum measurement. (d) EELS spectrum of silicon from silica nanoparticles in the associated area in (c).

Next, we evaluated how the detected signals in the elemental mapping of silica nanoparticles depend on particle size (Figure 4). For each ROI, a zero-loss image was first acquired, followed by cryo-EELS imaging at a low dose rate without extra doses, and image analysis using the three-window method combined with image alignment across different energy ranges (Figures 1 and S3). This approach enabled accurate background estimation, leading to reliable elemental mapping even from noisy images taken at 25k magnification. In contrast, simple background subtraction using the two-window method failed to produce reliable elemental mapping (Figures S5c,d) likely due to the high level of plasmon loss in ice (Figure 2). The EL images processed using the three-window method showed that the silicon signal was clearly visualized for 50 and 30 nm nanoparticles, demonstrating successful elemental mapping of individual silica particles (Figure 4a,b,d,e). However, when comparing the particle size distribution calculated from the zero-loss images, we observed that for particles approximately 15 nm or smaller, noise levels increased significantly (Figures 4c,f and S6), making particle morphology less distinguishable in cryo-EL images. Nevertheless, signal enhancement through binning and Gaussian blurring revealed corresponding particles between the zero-loss and Si-L_2,3_ core-loss images (Figure 4g–j). Some mismatches may be attributed to weak signals form insufficient particle thickness and thick ice, or to drifts across exposures at different energy windows. Using eq 5, the ice layer thickness was estimated to be approximately 60 nm based on the ratio of the unfiltered image intensity to the zero-loss intensity in Figure 4c. These observations suggest that the proposed method has a detection limit for particle sizes around 10 nm, which, to our knowledge, represents the highest spatial resolution achieved for elemental mapping of liquid-phase materials.^26,27^

**Figure 4:**
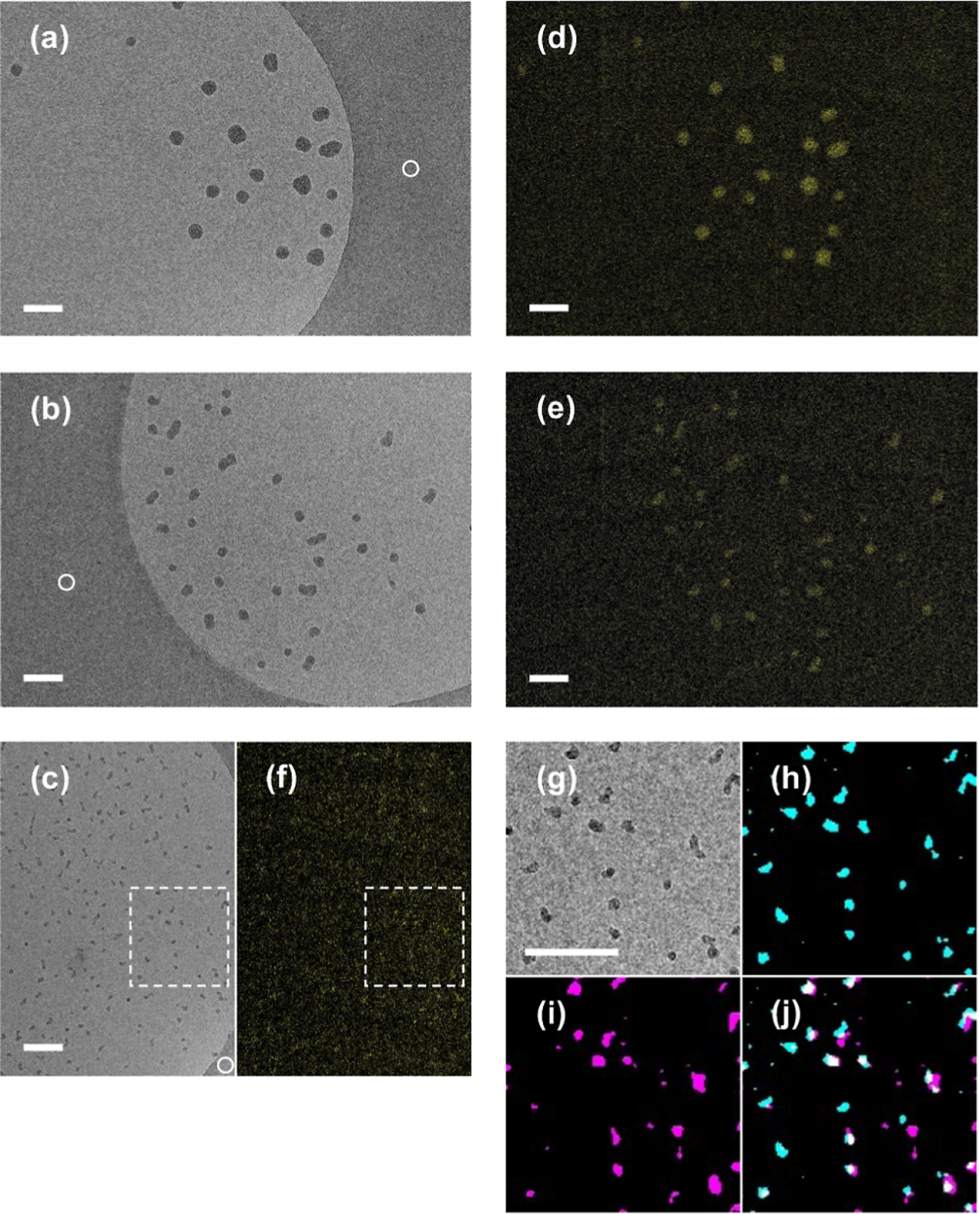
Elemental mapping of various sizes of silica nanoparticles dispersed in vitrified ice. (a–c) Zero-loss images of silica nanoparticles with 50, 30, and 10 nm diameter. The areas with white circles indicate carbon support film. (d–f) Cryo-EL images generated by the three-window method. Si-L_2,3_ core-loss signals are colored in yellow in (d–f). (g–j) Magnified views of the dashed boxes in (c,f). The magnified zero-loss image (g) was binarized and pseudocolored in cyan (h), while the corresponding boxed region of the Si-L_2,3_ core-loss image (f) was binarized and pseudocolored in magenta (i). Overlaps of cyan points in the zero-loss image and magenta points in the Si-L_2,3_ core-loss image are shown in white in (j). Scale bars: 100 nm.

### Simultaneous Mapping of Silicon and Carbon in Protein-Coated Nanoparticles

Given the successful elemental mapping of silica nanoparticles, we further investigated the detection of elemental signals from biological samples, such as proteins, to assess potential applications in biomaterials. For this purpose, 100 nm silica nanoparticles coated with streptavidin were used (Figure 5a). Streptavidin-coated nanoparticles possess biotin-binding capability, and the streptavidin–biotin interaction is widely utilized in biochemical applications. The streptavidin coating is presumed to be a monolayer approximately 5–6 nm thick; however, the boundary between the nanoparticles and the streptavidin could not be clearly distinguished in the zero-loss image. To simultaneously detect signals from both core silica particle and the protein coating, EELS measurements were performed to identify silicon and carbon signals. Signal acquisition was conducted in a hole region with highly dispersed nanoparticles using an SA aperture (Figure 5b,c).

**Figure 5:**
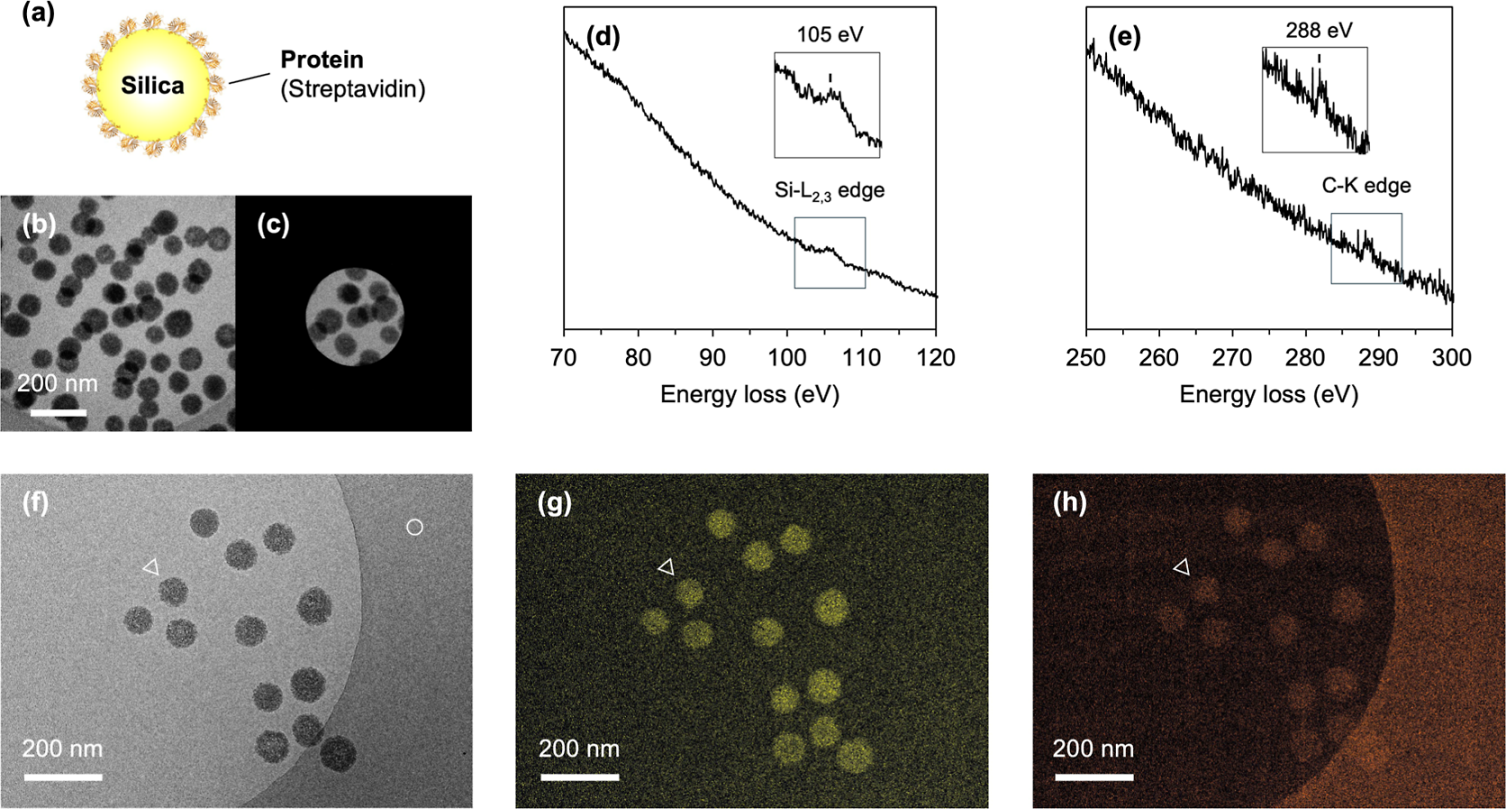
EELS spectra and cryo-EL images of protein-coated silica particles. (a) Schematic depiction of the streptavidin-coated silica particles. (b) Zero-loss image of the nanoparticles dispersed in vitrified ice. (c) Selected area in (b) for EELS spectra measurement. (d) EELS spectrum of Si-L_2,3_ edge. (e) EELS spectrum of C–K edge. (f) Zero-loss image of the nanoparticles in vitrified ice. (g) Cryo-EL image of the Si-L_2,3_ edge. (h) Cryo-EL image of the C–K edge. The Si-L_2,3_ and C–K core-loss signals are colored in yellow and orange, respectively. White circle and triangles indicate the carbon support film and one nanoparticle, respectively.

As with unmodified silica nanoparticles, applying an ES to the silicon energy loss region enabled the detection of the Si-L_2,3_ edge, originating from the nanoparticle core (Figure 5d). Subsequently, spectral acquisition was performed by applying an ES to the carbon energy loss region within the same ROI. As a result, the C–K edge signal, attributed to carbon from the protein bound to the silica particle surface, was successfully detected, although it was significantly weaker than the silicon signal (Figures 5e and S7). This difference in signal intensity is likely due to the lower spectral peak values at the C–K edge (Figures 5e and S7) and the relatively smaller size of the streptavidin tetramer, which has a diameter of approximately 5–6 nm, significantly smaller than the 100 nm diameter of the silica nanoparticles.

Next, we examined the simultaneous acquisition of cryo-EL images for silicon and carbon within the same ROI. After acquiring a zero-loss image (Figure 5f), elemental mapping of silicon was conducted using the three-window method. Consistent with the observation in Figure 4, the nanoparticles were clearly detected, allowing for the visualization of both silicon and carbon distributions (Figure 5g,h). These results suggest that this method can be applied to the simultaneous visualization of coating coverage and the dispersion state of modified nanoparticles. Streptavidin forms a tetramer with a molecular weight of 53 000 Da, and each monomer consists of a polypeptide chain of approximately 127 amino acids, classifying it as a relatively small protein. The ability to detect surface modification of nanoparticles by such a small protein facilitates the evaluation of functional materials derived from these modifications and enables the potential detection of organic contaminations.

### Detection of Phosphorus and Calcium from Solvent-Dispersed Particles

To expand the detection capabilities for other elements commonly found in biomaterials, we conducted cryo-EELS analyses for hydroxyapatite (HAp) particles, which do not contain silicon or carbon but instead consist of by phosphorus and calcium.^42,43^ HAp is an inorganic compound composed of Ca_10_(PO_4_)_6_(OH)_2_, which serves as the primary structural component of human bones and teeth and is widely utilized in life science, including column packing material for chromatography. Also, phosphorus is a key component of nucleic acids, while both phosphorus and calcium play essential roles in fundamental biological processes such intracellular signal transduction, muscle contraction, nerve activity, blood clotting, and enzyme functions.

Spectral measurements of phosphorus and calcium were initially performed for a ROI containing HAp particles (Figure 6a,b). By applying an ES to the phosphorus and calcium energy loss regions, we successfully detected the signals, the P-L_2,3_ edge and Ca-L_3_ and Ca-L_2_ edges (Figure 6c,d). Similar to the Si-L_2,3_ edge observed in Figure 3, the P-L_2,3_ edge appeared embedded within the water plasmon signal (Figure 6c). In contrast, the Ca-L_3_ and Ca-L_2_ edges were sharply detected (Figure 6d) despite their low absolute signal intensity (Figure S8).

**Figure 6:**
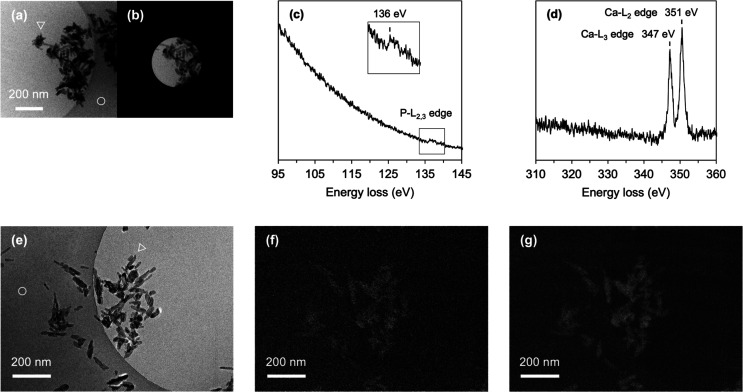
EELS spectra and cryo-EL images of hydroxyapatite (HAp). (a) Zero-loss image of HAp nanoparticles dispersed in vitrified ice. (b) Selected area of (a) for EELS spectra measurement. (c)­EELS spectrum of the P-L_2,3_ edge from (b). A box around 136 eV corresponds to the P-L_2,3_ core-loss peak and is shown in a zoomed-in view in the inset. (d) EELS spectrum of the Ca-L_2,3_ edge. (e) Zero-loss image of nanoparticles in vitrified ice. (f) Cryo-EL image of the P-L_2,3_ edge. (g) Cryo-EL image of the Ca-L_2,3_ edge. Both P-L_2,3_ and Ca-L_2,3_ core-loss signals are colored in white. White circles and triangles in (e) indicate the carbon support film and HAp nanoparticles, respectively.

Since the EEL signals from phosphorus and calcium were successfully detected, we proceeded with cryo-EL images. In the zero-loss image, HAp particles appeared more aggregated and exhibited lower dispersibility (Figure 6a,e) compared to the silica nanoparticles (Figures 4a–c and 5f). EELS imaging using the phosphorus and calcium signals identified their distribution within the HAp nanoparticles (Figure 6f,g). The detected phosphorus and calcium signals precisely matched the positions of HAp nanoparticles in amorphous ice and on the carbon support film, as observed in the zero-loss image. However, the P-L_2,3_ core-loss image exhibited a higher noise level. To evaluate the detection levels of each element, we calculated the signal intensity of phosphorus and calcium relative to the background (Figure S9). The signals in the cryo-EL images for the same ROI were estimated to be 1.47 for phosphorus and 2.52 for calcium. This is consistent with previous findings, where the Ca-L_3_ and Ca-L_2_ distinct peaks were clearly detected for calcium, while the P-L_2,3_ peak appeared weaker for phosphorus.^44^ In addition, signals from the carbon film were visible in the contrast-enhanced EL image along with the calcium signals (Figure S9e). This is likely due to the relatively close energy loss values between carbon and calcium, resulting in a shoulder effect from the carbon peak. However, since the calcium-derived signal was significantly stronger than that from the carbon film, it is considered to originate specially from calcium at the defined energy loss. In contrast, the carbon film was not visible in the P-L_2,3_ core-loss image, as the P-L_2,3_ and C–K edges are well separated in energy (Figure S9d). These results highlight the strong potential of cryo-EELS to advance applications in biomaterials research.

However, the acquired signals were relatively weak, and the total dose required for signal acquisition ranged between 910–920 e^–^/Å^2^. While this is less than several percents of the irradiation dose typically used in noncryogenic EELS analyses of materials,^19,20,45^ it remains relatively high in cryo-TEM and approaches the threshold for maintaining the amorphous ice film. Therefore, no additional low-loss spectra were acquired for analyzing and removing multiple plasmon scattering in each ROI. Moreover, biomaterials are even more sensitive to electron irradiation, with structural damage occurring at doses ranging from a few to several tens of electrons per Å^–2^. While significant gaps remain in applying single-particle cryo-EM elemental mapping of biological samples, the proposed approach successfully balances accurate and reliable elemental mapping of liquid-phase materials with electron dose.

## Conclusions

In this study, we developed an elemental mapping method for silica and HAp nanoparticles dispersed in frozen solvent by integrating cryo-TEM with a highly coherent electron beam, an energy filter, and a high-sensitivity direct electron detector. Cryogenic data acquisition minimized radiation damage while enabling higher-resolution elemental mapping of specimens embedded in amorphous ice. Dose-fractionated data collection further improved image quality through precise drift correction during extended exposure times,^30,31^ achieving elemental mapping with a spatial resolution of 10 nm—one of the highest reported for elemental mapping of nanoparticles in solvent.

Moreover, we successfully detected multiple elements within the same ROI in ice-embedded sample, demonstrating the ability to visualize biochemically modified materials and determine their elemental compositions. The high-resolution detection of biologically relevant light elements, such as phosphorus, calcium, and carbon, highlights the potential of cryo-EELS for analyzing biological samples, including nucleic acids and proteins. Furthermore, cryo-EELS enabled reliable elemental identification of hydrated soft materials composed of light elements, allowing for visualization of both structural and elemental distributions within finely controlled microenvironments, such as surface modifications.

Moving forward, this technique can be extended to a wide range of soft materials, including carbon-based materials, drug delivery systems, biomedical materials, and food science. These advancements are expected to significantly contribute to research in both life and materials sciences, opening new possibilities for high-resolution elemental analysis in hydrated environments.

## Supplementary Material



## Notes

Additional experimental details including a GUI of EnergyShift in ParallEM, raw EELS spectra, a flow diagram of image acquisition, scale-adjusted cryo-EELS spectra, elemental maps generated by two-window method, size distribution of silica nanoparticles, and analysis of signal intensity in the cryo-EL images of HAp (PDF)
