# Supplementary material for: Low-Dose Elemental Mapping of Light Atoms in Liquid-phase Materials Using Cryo-EELS
Source: Anal Chem. 2025 Jul 31;97(33):18055–63. doi: 10.1021/acs.analchem.5c02121 (PMC12392256; doi:10.1021/acs.analchem.5c02121)
Supplement: Supplementary file 1 [file ac5c02121_si_001.pdf]

Supporting Information

Low-Dose Elemental Mapping of Light Atoms in Liquid-phase Materials Using Cryo-EELS

Daisuke Unabara[a], Yohei K. Sato[a], Tasuku Hamaguchi[a, *], and Koji Yonekura[a, b, *]

[a]Institute of Multidisciplinary Research for Advanced Materials (IMRAM), Tohoku University, 2-1-1 Katahira, Aoba-ku, Sendai, Miyagi, 980-8577, Japan

[b]Biostructural Mechanism Laboratory, RIKEN SPring-8 Center, 1-1-1 Kouto, Sayo, Hyogo, 679-5148, Japan

* Correspondence to: koji.yonekura.a5@tohoku.ac.jp; tasuku.hamaguchi.c3@tohoku.ac.jp

Contents

Figure S1. A GUI of EnergyShift, developed to facilitate cryo-EELS. Included in ParallEM.

Figure S2. Raw EELS spectra recorded with the XF416ES detector.

Figure S3. Flow diagram to capture prepre-edge, pre-edge and core-loss images.

Figure S4. Scale-adjusted cryo-EELS spectra for the carbon support film in Figure 2(b) and vitrified ice in Figure 2(c).

Figure S5. Elemental maps generated by the two-window method.

Figure S6. Box plot of the size distribution of silica nanoparticles.

Figure S7. Scale-adjusted cryo-EELS spectra for 100 nm streptavidin-coated silica particles in vitrified ice.

Figure S8. Scale-adjusted cryo-EELS spectra for HAp nanoparticles in vitrified ice.

Figure S9. Analysis of signal intensity in the cryo-EL images of HAp.

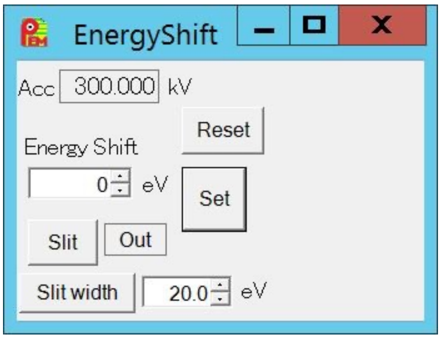

Figure S1. A GUI of EnergyShift, developed to facilitate cryo-EELS. Included in ParallEM.

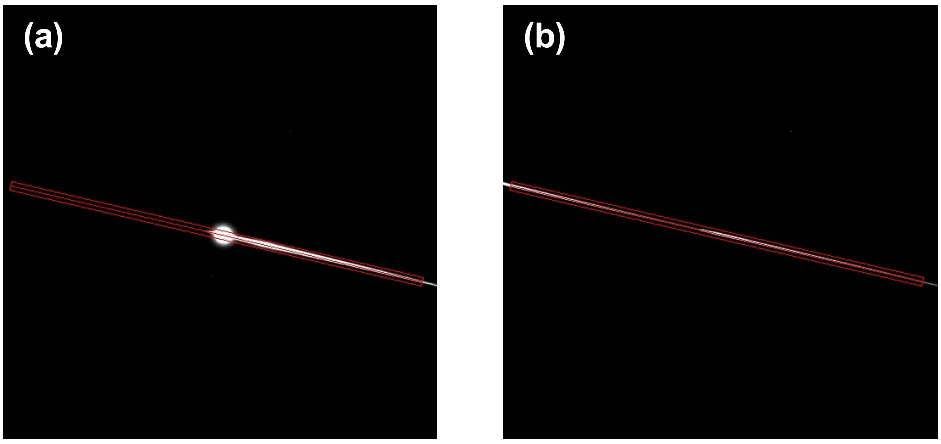

Figure S2. Raw EELS spectra recorded with the XF416ES detector. (a) Corresponding to the zero-loss peak and low-energy loss region shown in **Figure 2(a)**. (b) Corresponding to the core-loss region shown in **Figure 2(b)**. The red lines along the energy loss spectrum indicate the spectral center and the edges defining the region where intensity is summed across the center line.

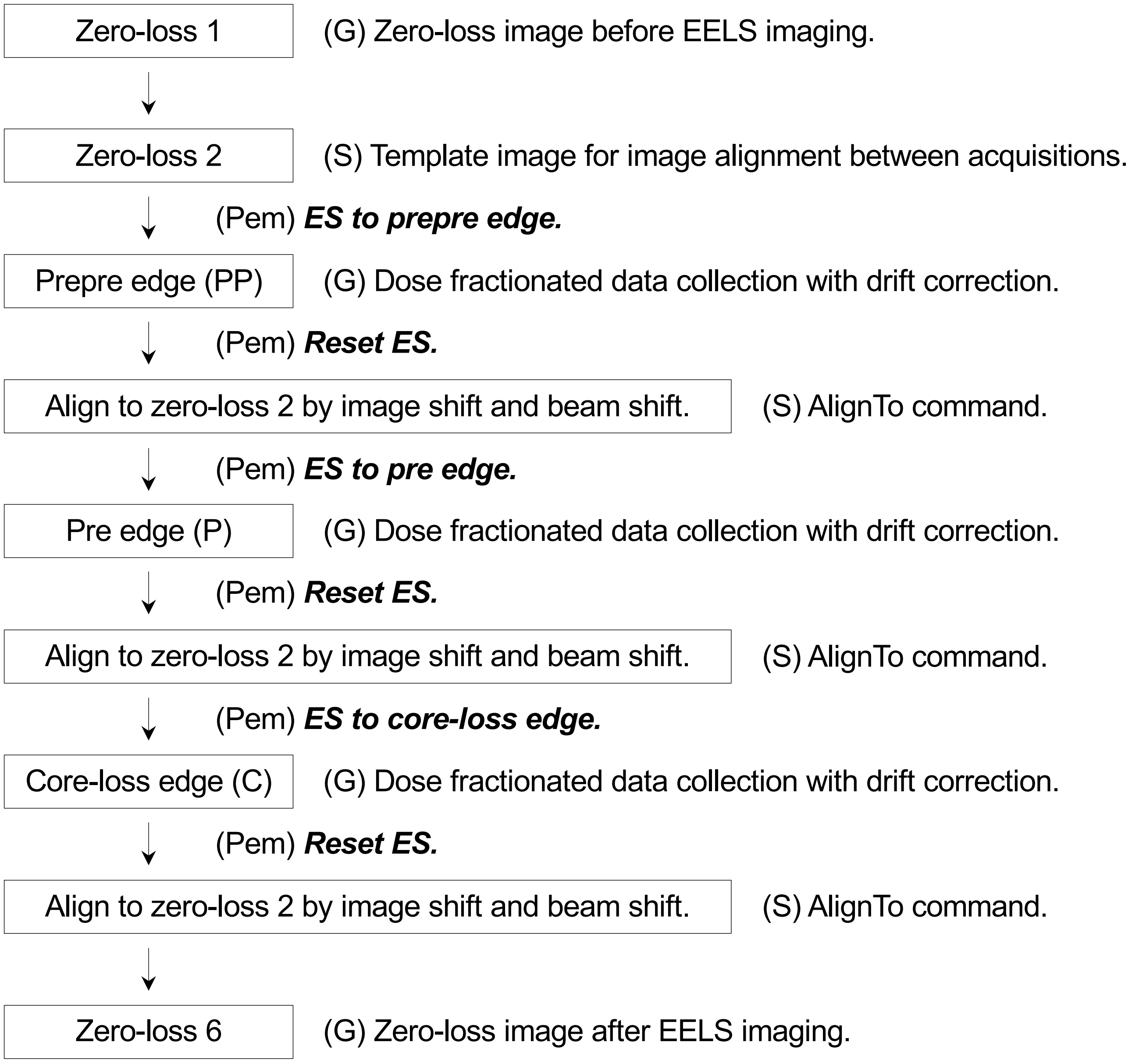

Figure S3. Flow diagram to capture prepre-edge, pre-edge and core-loss images. ES: Energy shift. (G), (S) and (Pem) indicate operations through DigitalMicrograph, SerialEM and ParallEM.

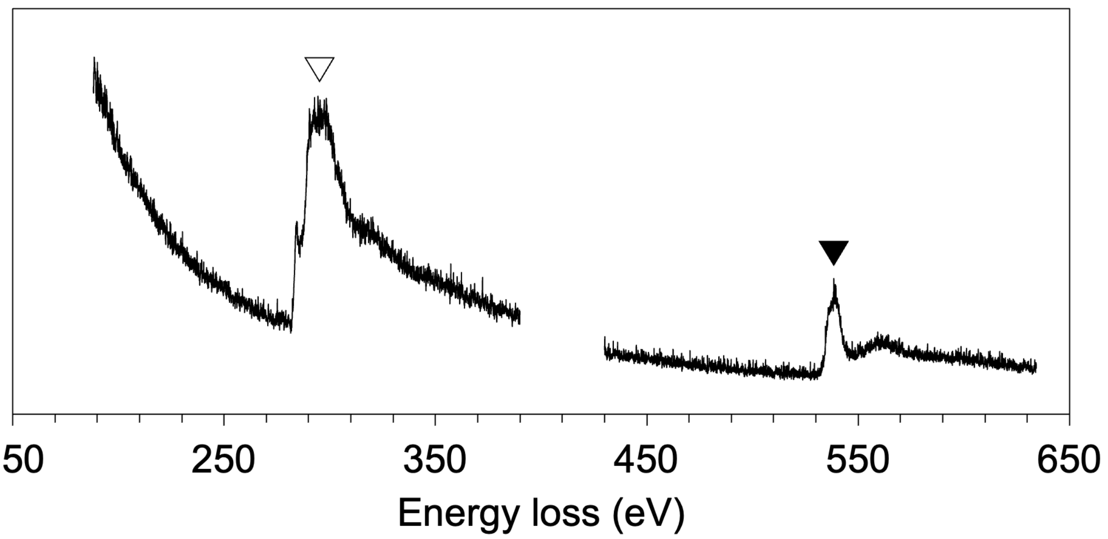

Figure S4. Scale-adjusted cryo-EELS spectra for the carbon support film in **Figure 2(b)** and vitrified ice in **Figure 2(c)**. Open and closed triangles indicate the C-K and O-K core-loss signals, respectively. The spectra from 190−390 eV and 430−630 eV were acquired independently from the same ROI.

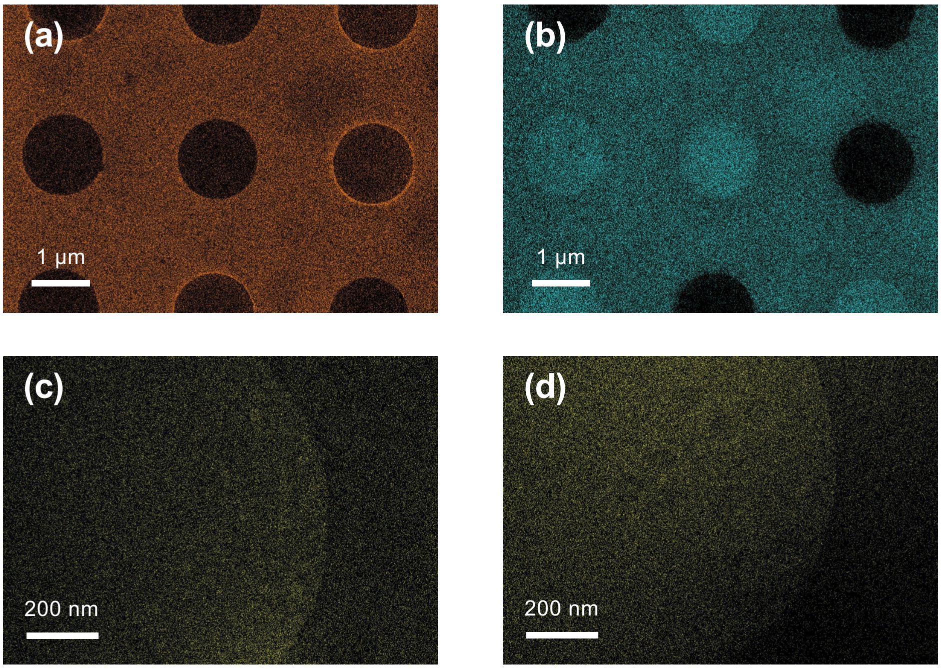

Figure S5. Elemental maps generated by the two-window method. (a) C-K core-loss image of the carbon support film as in **Figure 2(e)**. (b) O-K core-loss image of the vitrified ice as in **Figure 2(f)**. (c) Si-$L_{2,3}$ core-loss image of the 50 nm silica nanoparticles as in **Figure 4(d)**. (d) Si-$L_{2,3}$ core-loss image of the 100 nm the silica nanoparticles as in **Figure 5(g)**.

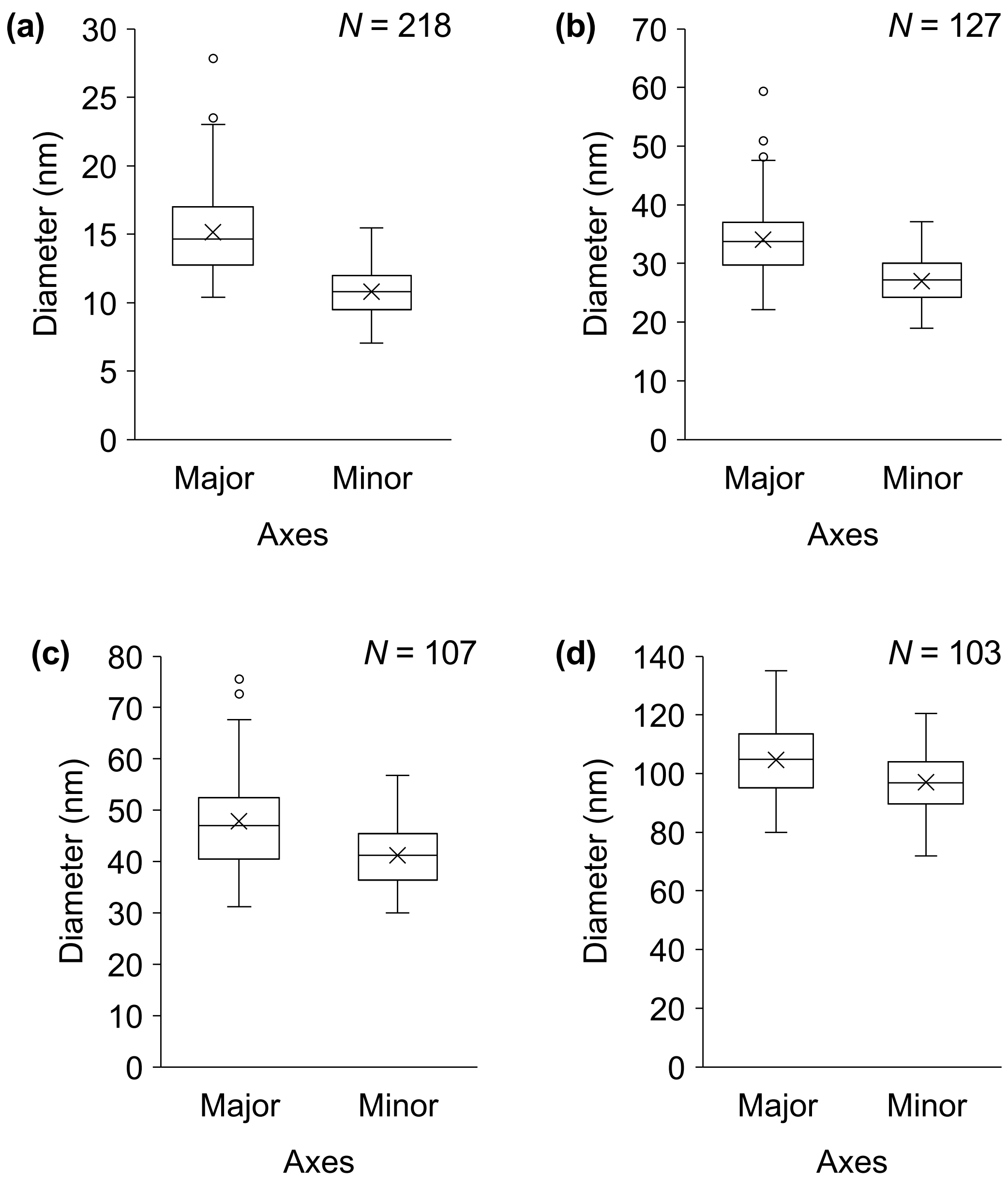

Figure S6. Box plot of the size distribution of silica nanoparticles. (a) 10 nm particles. (b) 30 nm particles. (c) 50 nm particles. (d) Streptavidin-coated 100 nm particles. X marks indicate the average values. Horizontal bars indicate minimum, lower quartile, median, upper quartile, and maximum from the bottom. White circles indicate outliers.

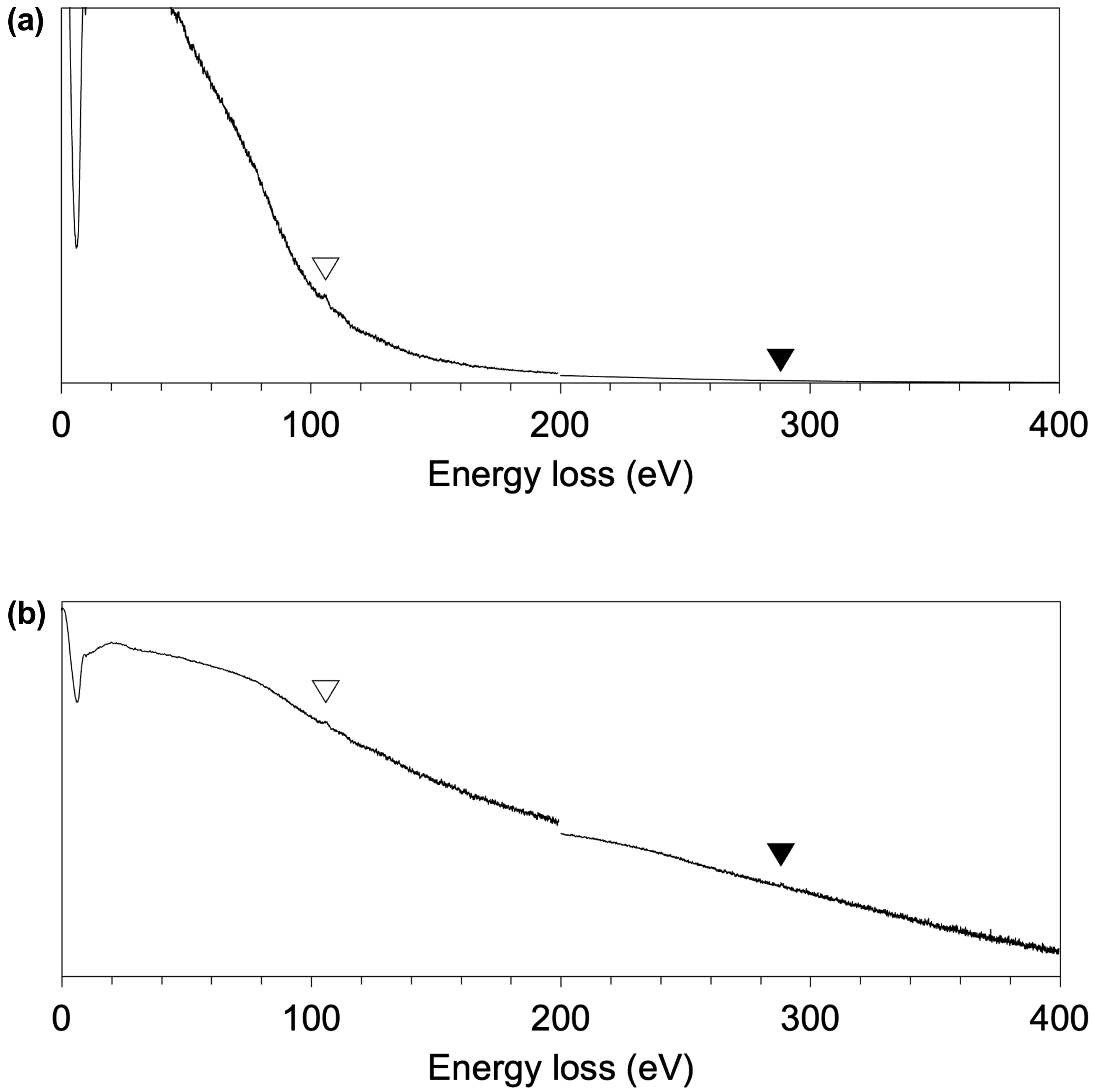

Figure S7. Scale-adjusted cryo-EELS spectra for 100 nm streptavidin-coated silica particles in vitrified ice. (a) Intensity plotted on a linear scale. (b) Plotted on a logarithmic scale. Open and closed triangles indicate the Si-$L_{2,3}$ and C-K core-loss signals, respectively. The spectra from 0−199 eV and 200−400 eV were acquired independently from the same ROI.

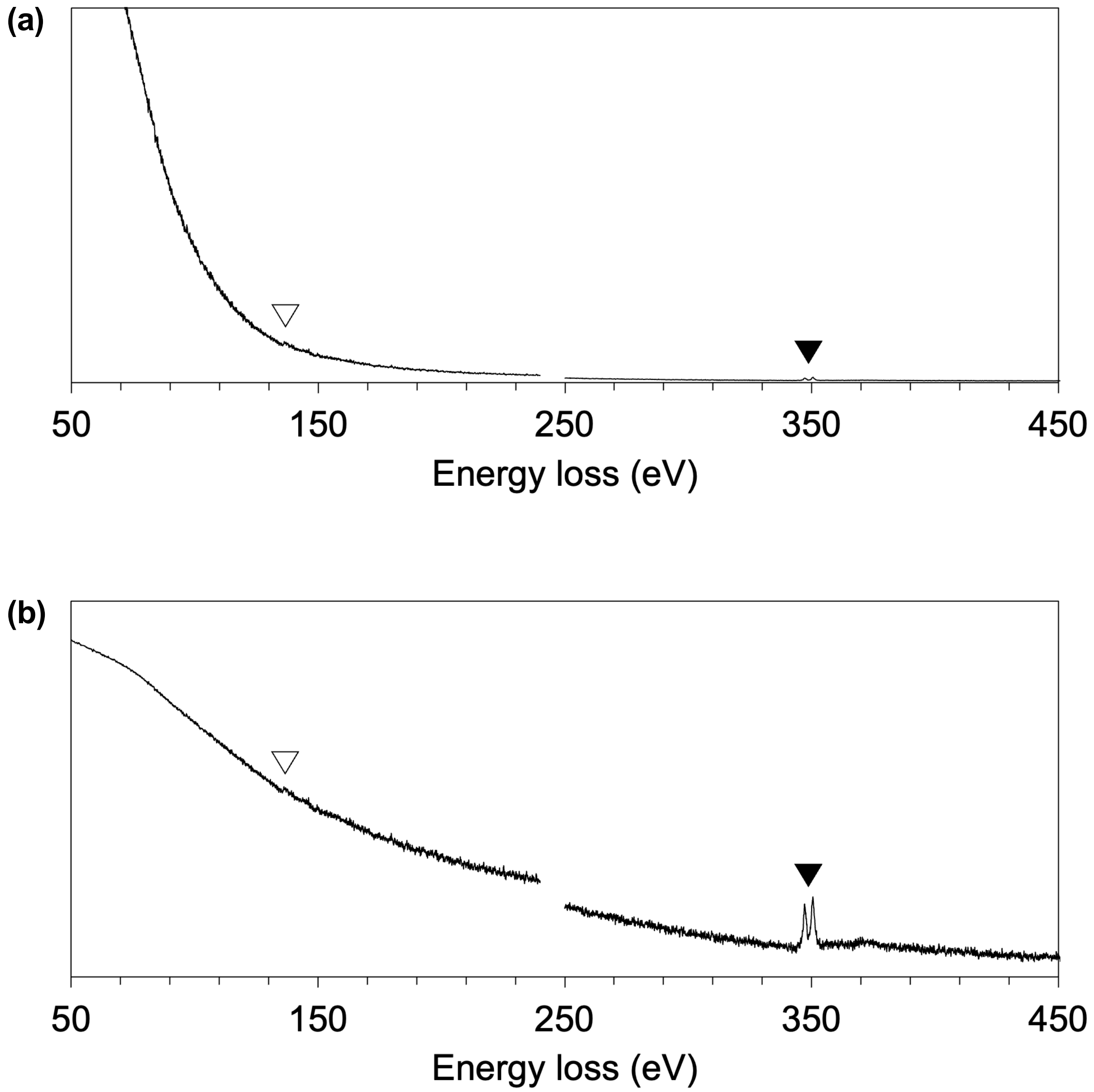

Figure S8. Scale-adjusted cryo-EELS spectra for HAp nanoparticles in vitrified ice. (a) Intensity plotted on a linear scale. (b) Plotted on a logarithmic scale. Open and closed triangles indicate the P-$L_{2,3}$ and Ca-$L_{2,3}$ core-loss signals, respectively. The spectra from 50−240 eV and 250−450 eV were acquired independently from the same ROI.

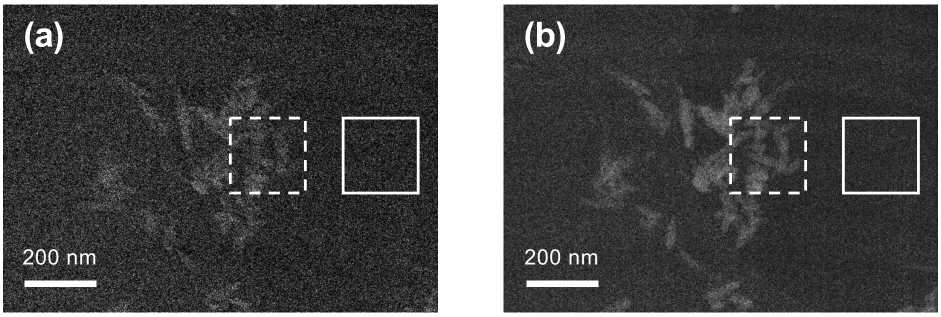

(c)

	Region	Ratio of the mean values in the regions with and without nanoparticles	Relative Intensity
P-$L_{2,3}$ core-loss image	With HAp nanoparticles	4.73	1.47
	Without nanoparticles	3.22	
Ca-$L_{2,3}$ core-loss image	With HAp nanoparticles	3.20	2.52
	Without nanoparticles	1.27	

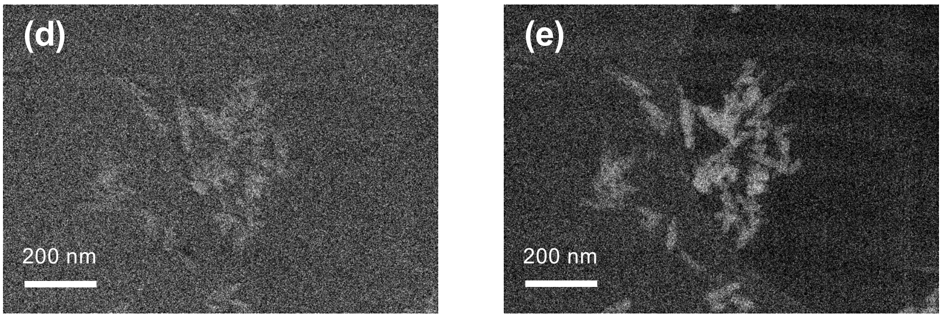

Figure S9. Analysis of signal intensity in the cryo-EL images of HAp. (a) The P-$L_{2,3}$ core-loss image as in **Figure 6(f)**. (b) The Ca-$L_{2,3}$ core-loss image as in **Figure 6(g)**. White dashed and solid boxes in (a) and (b) enclose the same area, including HAp nanoparticles and the ice region without nanoparticles, respectively. (c) Intensity within the box areas. The mean intensity values within the dashed boxes are 4.73 and 3.20 for the P-$L_{2,3}$ and Ca-$L_{2,3}$ core-loss images, respectively, while the ratios of the mean values within the dashed boxes to those within the solid boxes are 1.47 and 2.52, respectively. (d) The same image as in (a) but shown with enhanced contrast. (e) The same image as in (b) but shown with enhanced contrast.
